# Effects of system conditions on growth performance reproductive efficiency, sex hormones and seeds production of African catfish, *Clarias gariepinus*

**DOI:** 10.1186/s12917-026-05630-5

**Published:** 2026-06-30

**Authors:** Hamed H. E. Saleh, El-Sayed I. Attia, Rasha K. Abdel-Wahed, Gad H. G. Hussein, Mohamed S. Hassaan, Mohamed R. Soaudy

**Affiliations:** 1 Aquaculture Division, National Institute of Oceanography and Fisheries (NIOF), Cairo, Egypt; 2 Department of Animal Production, Faculty of Agriculture, Cairo University, Cairo, Egypt; 3 Department of Animal Production, Faculty of Agriculture at Moshtohor, Fish Research Laboratory, Benha University, Benha, 13736 Egypt

**Keywords:** Catfish, Biofloc technology, Spawning, Reproduction efficiency and larval production

## Abstract

Biofloc technology (BFT) is an aquaculture practices which rely on maintaining a minimum-water exchange throughout the culture period, presenting a promising solution to enable a more sustainable and eco-friendly approach to fish farming. The present study examined the effect of different rearing conditions (three treatment with three replicates):first treatment non-water exchange (NWE) system; without carbon source addition and without water exchange, second treatment, clear water (CW) system: without carbon source addition and with 50% water exchange weekly, and third treatment, biofloc technology (BFT) system; addition of molasses as carbon source daily without water exchange on the growth performance, blood biochemical parameters, sex hormones, reproduction efficiency and larval production of catfish brood-stock. Two phases of brood-stock were examined under the different rearing conditions including grow out phase and spawning phase. For the grow-out phase immature catfish males and females were distributed in concrete ponds (2 × 2 × 1 m^3^). Each pond stocked with 15 fish (8 females and 7 males) at stocking rate 5 fish m^−1^. Fish were fed at a rate of 2% of body weight/day, with a commercial diet containing 30% crude protein during the experimental period that continued for 120 days. For spawning phase brood fish were stocked in the same ponds and the same treatments at stocking rate of 4 brood-stock pond^−1^ (2 females and 2 males). The best water quality conditions, the highest growth parameters values, the better Absolute fecundity (AF), relative fecundity (RF), gonado somatic index (GSI), and egg diameters were recorded in BFT system compared to NWE and CW systems. The best blood biochemical parameters (cortisol, glucose, cholesterol, creatinine and urea) were recorded in BFT system. The superior larvae parameters were recorded in BFT system compared to NWE and CW systems. In conclusion biofloc technology is an appropriate condition for rearing African catfish where it has a positive effect on growth and reproductive performance of the brood fish.

## Introduction

Aquaculture has been the fastest-growing source of food globally over the last thirty years, and it currently supplies over half of the world's fish consumption needs [43, 69, 87, 110]. It also contributes significantly to food security, income generation, and economic prosperity in communities [9, 51]. In recent years, maintain sustainable aquaculture systems are necessary to increase the fish production [27, 30, 56, 88]. There is a growing drive for intensification in aquaculture to meet the rising demand for several aquatic products, meanwhile elevated stocking densities and feed inputs require repeated water exchanges to uphold water quality in traditional systems [24]. Forming innovative systems that require little to no water exchange, especially in areas facing water scarcity as the case in Egypt is a crucial step toward promoting sustainable growth in aquaculture.

Biofloc technology (BFT) is an aquaculture practice which rely on maintaining a minimum-water exchange throughout the culture period [4, 85], presenting a promising solution to enable a more sustainable and eco-friendly approach for fish farming [22]. It can be done by the addition of carbohydrate source to regulate the balance of carbon and nitrogen components [13, 21, 54]. This additional supplementation promotes the proliferation of naturally existing heterotrophic bacteria and chemoautotrophic bacteria, which control toxic nitrogenous compounds in the water [11, 12, 20, 26, 101]. The Bioflocs serve two essential functions within BFT aquaculture systems: firstly, they eliminate ammonia produced by the excretion of cultivated animals and decomposition of feces and/or uneaten food,secondly, they potentially offer nutrition to aquaculture species through consumption of bioflocs [25, 55].

African catfish (*Clarias gariepinus*) is a freshwater fish species extensively cultivated for consumption in Africa, as well as various regions across the world [72]. It holds significant promise for development due to its rapid growth, capacity to utilize various food sources, resilience to varying water conditions, and suitability for cultivation at remarkably high densities [59]. However, one of the challenges that catfish farming faces is the residual aquaculture waste which yields significant quantities of nitrogen and carbon and can negatively affect the environmental capacity [109]. The last challenges in catfish farming can be resolved by the application of biofloc through convert the wastes to useful product and enhance water quality [79].

Previous studies have indicated that African catfish cultivate successfully in biofloc systems [22, 24, 25, 35, 44, 53, 117]. Application of biofloc technology can reduce water usage during the production of catfish, *C. Gariepinus* Dauda et al. [22]. This technology has the potential to effectively preserve water quality, minimize water consumption and waste generation, offer a nourishing source, enhance feed utilization efficiency, reduce feed expenses, and boost productivity [16, 38]. Moreover, previous research confirmed that implementing biofloc systems not only enhanced feed efficiency and decreased nutrient waste, but also had positive impacts on the immunity [19, 33, 67, 116] and the reproductive performing of the cultivated organisms [18, 19, 34, 37]. Fish maturation and spawning adversely affected by the environmental conditions especially water quality, while larvae of fish needs a source of natural food after hatching [83]. Incidentally, optimal water conditions and the plentiful presence of bioflocs as a live nutrients source for larvae within biofloc systems have the potential to boost the success of catfish brood-stock reproduction. In this regard few studies examined the reproduction performance of catfish under biofloc system like Ekasari et al. [35] where they found biofloc significantly affected broodstock and larval rearing of African catfish. As authors knowledge the data regarding the reproductive performance of brood-stock under BFT condition is still limited, especially when compared to nursery and grow-out phases. Therefore, the present study objectives were to evaluate the effects of biofloc technology (BFT) application on the growth performance, blood biochemical parameters, sex hormones, reproduction efficiency and larval production of African catfish (*Clarias gariepinus*) brood-stock in nursery and grow-out phases.

## Material and methods

### Ethics approval

This work was conducted with the strict recommendations and approval of the National Institute of Oceanography and Fisheries (NIOF, Egypt) Committee for ethical care and use of animals/aquatic animals (NIOF-IACUC, Code: NIOF-AQ5-F-24-R-019). Moreover, all study methodologies rigorously adhered to the ARRIVE guidelines v2.0 [92], ensuring that the research approach is consistent with recognized ethical standards and preserves the wellbeing of the experimental animal.

### Experimental design

One-way experiment was designed to evaluate the effect of three different rearing conditions: the first system is non-water exchange (NWE) system (without any addition and without water exchange, the second system is clear water (CW) system (without any addition but 50% water exchange weekly and the third system is biofloc technology (BFT) system (using molasses as carbon source and without water exchange on the growth performance and reproduction efficiency of brood-stock catfish. A C: N ratio of 10:1 was used in this experiment according to De Schryver et al. [26] and Soaudy et al. [103] for biofloc start up. The molasses was weighed, dissolved in water and added daily to biofloc ponds in a split-order manner at in-between feeding times according to De Schryver et al. [26] and El–Husseiny et al. [29]. The experimental design was carried out in two phases,grow out phase and spawning phase.

### Phase Ӏ: grow-out phase of catfish brood-stock

Immature catfish males and females were obtained from the commercial private fish farm in Kafer El-Shakh Governorate, Egypt. Fish were selected according to similar weight, good health and free of malformations and lesions. Fish transported to commercial hatchery in Kafer El-Shakh Governorate and sterilized by placing in plastic tanks filled with formalin (0.15ml/10l of water, i.e. 15 ppm). After that the immature catfish males and females with an initial body weight 283 g ± 2.82g for male and 360 g ± 2.82g for female were distributed in nine concrete ponds (2 × 2 × 1m^3^) three treatment with three replicates. Each pond was stocked with 15 fish (8 females and 7 males) at stocking rate 4 fish m^3^. Fish were fed at a rate of 2% of body weight/day, with a commercial diet with proximate composition (30% crude protein,5% lipid, 4.55 fibre and 4,000 kcal/kg) during the experimental period that continued for 120 days. Ponds were filled with fresh water and water exchange by 50% was done twice weekly in CW system, while no water exchange was performed in biofloc and NWE systems except for the compensation of evaporated water. Ponds were aerated by air blower. At the end of the grow-out phase fish were weighted and counted to determine growth and survival rate. Eight fish (4 males and 4 females) from each treatment were collected for blood sex hormone and blood biochemical analysis. Also, four catfish females were selected and sacrificed to determine fecundity, gonado-somatic index (GSI) and egg diameter.

### Phase ӀӀ: spawning phase

After grow-out phase (120 days) catfish males and females were reached to mature weight. Total of 36 mature males and females catfish were selected from grow out phase. The selected brood fish were injected with human chorionic gonadotropin (hCG) hormone at a rate of 3,000 IU/kg for female and 1500 IU/kg for male according to Attia et al. [10]. Brood fish were stocked in the same ponds and the same treatment (NWE, CW and BFT) at stocking rate of 4 brood-stock per pond (2 females and 2 males) with an average weight 631 g ± 5.61, 717.5g ± 5.6

1 and 762 g ± 5.61 for male and 680 g ± 5.61, 743 g ± 5.61 and 799 g ± 5.61 for female from different treatments; NWE, CW and BFT, respectively. The ponds were supplemented with ornamental palm leaves to offer a surface for the fish to lay their eggs.

### Rearing of larvae

After hatching the larvae were counted and stocked in glass aquaria (20 L volume). The glass aquaria filled with water taken from the water ponds of reared brood fish. larvae were stocked in glass aquaria at 10 larvae/aquaria and reared under the same system condition in brood-stock experiment, NWE system, clear water (CW) system and biofloc technology (BFT) system. The larvae fed with commercial powder feed with 40% crude protein at a rate of 15% from biomass four times a day (8:00 am, 11:00 am, 2:00 pm, and 5:00 pm) for 20 days. Glass aquaria were aerated by air blower for oxygen requirement.

Baker’s yeast (*Saccharomyces cerevisiae*) was added to all fry ponds after solving one gram of yeast in one litre of water, it’s important to fry according to Abdel-Tawwab et al. [2].

### Water quality parameters

Water parameters including pH and dissolved oxygen were measured weekly by Tintometer group® (pH/OPR, DO, CD/TDS, Nr: 00724200. Germany 01/16), while temperature assessed every day by centigrade thermometer. Spectrophotometer model (LKB Bichrom UV visible spectrophotometer) was used to determine water ammonia nitrogen, nitrite and nitrate every two weeks according to the method by APHA [7]. Floc volume was measured using Imhoff cone as the volume of floc on the bottom of the cone was measured after 15 min of sedimentation [12]. Total suspended solids (TSS) was determined by calorimetrically measurement of different water parameters and done using Spectrophotometer model (LKB Bichrom UV visible spectrophotometer) according to Grasshoff [50]. Total suspended solids or sludge was precipitated and removed when floc volume reached to 30 ml/L or TSS exceeded 400 mgL^−1^.

### Growth and feed utilization parameters

- Weight gain (g) = final weight, g—initial weight, g.
- Specific growth rate (%/day) = [(ln final weight—ln initial weight)/period in days] × 100, where ln is the natural log.
- Feed conversion ratio (FCR) = dry feed intake, g/weight gain, g.
- Survival rate (SR) % = (No. of fish survived at the end of the experiment/whole number of fish at the beginning) × 100.

### Biological parameters and reproductive performance of brood-stock

Gonad somatic index (GSI %) = [gonads weight (g)/body weight (g)] × 100.

Fecundity was estimated by counting the number of mature ova from a known weight of mature/ripe ovary. The subsamples were spread evenly on a counting slide with a few drops of water and the number of mature ova was counted to determine the fecundity by the following formula [1, 86, 95, 104]:

$$\begin{aligned} \mathrm{Absolute}\;\mathrm{fecundity}&=\;\mathrm{No}.\;\mathrm{of}\;\mathrm{ova}\;\mathrm{in}\;\mathrm{the}\;\mathrm{subsample}\\&\;\times\;\mathrm{total}\;\mathrm{ovary}\;\mathrm{weight}/\\&\;\mathrm{Weight}\;\mathrm{of}\;\mathrm{subsample} \end{aligned}$$

$$\begin{aligned} \mathrm{Relative}\;\mathrm{fecundity}&=\;\mathrm{Absolute}\;\mathrm{fecundity}\;/\\& \;\mathrm{total}\;\mathrm{weight}\;\mathrm{of}\;\mathrm{fish} \end{aligned}$$

### Blood parameters

Eight brood fish (4 males and 4 females) from each treatment were collected randomly for blood sex hormone and blood biochemical analysis. Fish were anesthetized by using clove oil (5 ml/l). Blood samples were drawn from the caudal vein of both males and females and subsequently transferred to Wasserman tubes. For determine the sex steroid hormones by enzyme-immunoassay technique, commercial kits of ELISA were used: (Cat. No: CSB-E13017Fh) was used for estrogen, (Cat. No: MBS933475) for testosterone and (P4-Cat. No11-PROHU-E01) for progesterone determination according to Rinchard et al. [97] and Alvarado et al. [5]. Enzymatic colorimetric method using BioSystem BTS-302 utilizing commercial kits of Bio diagnostic, Dokki, Giza, Egypt was used for determine the serum biochemical indices. Fischer et al. [45] and Mills et al. [84] validated the commercial kits of ELISA to determine the steroid hormones by running parallelism, accuracy and precision tests for each steroid and each fish species. Serum glucose, alanine aminotransferase (ALT), and aspartate aminotransferase (AST), were measured according to the method described by Trender [108], Reitman and Frankel [96], while creatinine and urea (mg/dl) were quantitatively assessed according to the method of Henry [62]. Cortisol levels (ng/ml) were determined by the fast quantitative method of fluorescence immunoassay (FIA) was used to measure the cortisol level, along with a commercial kit (Biodiagnostic Company, Egypt) [77].

#### Zooplankton samples

Zooplankton was collected from experimental tanks; 10 L of each water sample were filtered through plankton net 55 μ mesh size, 25 cm diameter and 80 cm length. Each sample collected was transferred to a labelled clean bottle and immediately fixed with 4% formaldehyde. After fixing, two millimeters of Rose Bengal stain (0.5%) were added. The filtrated samples were inspected under an optical research microscope using a Rafter cell with magnification varying from 100 to 400X. Based on the following equation, zooplankton was estimated after examining all of the identified species in each sample [8]: No of organisms/litter = N × D/S × C, Whereas N = Number of organisms for the calculated species, D = Volume of sample after filtration, and S = Number of subsamples. C = Total volume of the collected water sample.

#### Statistical analysis

Data were tested for homogeneity and normality tests before analyzed. Afterwards data were analyzed using one-way analysis of variance and the differences among means were made by using Duncan's multiple range test using SAS ANOVA procedure (SAS, version 6.03, Soft Inc., Tusla, OK, USA, [102]. The differences at *P <* 0.05 were considered significant. The values are presented as means ± standard error of the mean (SEM).

## Results

### Water quality parameters

Water quality parameters including, temperature, pH, DO, NH_4_, NO_2_, NO_3_, TSS, and floc volume of different treatments are listed in Table 1. All water quality parameters were within the appropriate range for catfish culture. BFT exhibited lower values of ammonia and nitrite and higher nitrate compared to CW and NWE. BFT and NWE had higher values of TSS and floc volume compared to CW.

**Table 1:** Water quality parameters for African catfish (*Clarias gariepinus*) under different rearing conditions

Parameters	Rearing systems			± SEM	*P*-value
	NWE	CW	BFT		
Temperature,°C	29.50	29.75	30.25	1.20	0.9082
pH	8.13^a^	8.20^a^	7.97^b^	0.03	0.0353
DO_,_ mg/l	7.15	7.05	7.10	0.43	0.9872
NH_3-_N_,_ mg/l	1.27^a^	0.81^b^	0.66^c^	0.03	0.0024
NO_2,_ mg/l	0.71^a^	0.64^b^	0.59^b^	0.01	0.0315
NO_3,_ mg/l	1.74^b^	1.92^ab^	2.05^a^	0.06	0.0873
TSS, mg/l	383.50^a^	169.50^b^	366.00^a^	10.97	0.0014
Floc volume, ml/l	4.09^b^	2.93^b^	21.00^a^	1.57	0.0071

Means followed by different letters in the same row are significantly different (*P ≤* 0.05)

*NWE* Non-water exchange system: fish were reared under unchanged water

*CW* Clear water system: water exchange rate of 50% of tank volume weekly

*BFT* Biofloc technology system: addition of molasses daily for tank and without water exchange

### Zooplanktons

Total count of different zooplankton species including, rotifer, protozoa, copepod in different treatments are displayed in Table 2. Significant differences (*P <* 0.05) were found in total count of different zooplankton species among different treatments. The highest total count of rotifer, protozoa, and copepod were recorded to BFT compared to CW and NWE.

**Table 2:** Total count zooplankton, number of individuals per liter under different rearing conditions

Parameters	Rearing systems			± SEM	*P*-value
	NWE	CW	BFT		
Rotifera	237.50^b^	885.00^b^	7,655.00^a^	170.9	0.0001
Protozoa	131.50^c^	271.00^b^	3,165.00^a^	27.00	0.0001
Copepoda	12.50^b^	38.00^a^	57.50^a^	4.52	0.0144
Total count zooplankton	381.50^b^	1,194.00^b^	10,877.50^a^	195.68	0.0001

Means followed by different letters in the same row are significantly different (*P ≤* 0.05)

*NWE* Non-water exchange system: fish were reared under unchanged water

*CW* Clear water system: water exchange rate of 50% of tank volume weekly

*BFT* Biofloc technology system: addition of molasses daily for tank and without water exchange

### Growth performance of males and females

Initial body weight (IBW), final body weight (FBW), weight gain (WG), specific growth rate (SGR), feed conversion ratio (FCR), and survival rate (SR) of both males and females reared under BFT system compared to NWE and CW systems are presented in Table 3. Males and females of catfish brood-stock reared under BFT system had higher values of FBW, WG, SGR, and SR than NEW and CW systems. The best FCR values for both males and females were recorded in BFT system.

**Table 3:** Fish growth parameters for African catfish (*Clarias gariepinus*) under different rearing conditions

Parameters	Rearing systems			± SEM	*P*-value
	NWE	CW	BFT		
Male					
IBW, g/fish	282.50	283.00	284.50	2.82	0.3972
FBW, g/fish	631.00^c^	717.50^b^	762.00^a^	5.61	0.0001
WG, g/fish	348.500^c^	434.50^b^	477.50^a^	3.17	0.0001
SGR,%/day	0.67^c^	0.78^b^	0.82^a^	0.002	0.0001
FCR	1.80^a^	1.65^b^	1.63^c^	0.008	0.0001
Female					
IBW g/fish	356.00	360.00	360.00	2.82	0.6804
FBW g/fish	680.00^c^	743.50^b^	799.00^a^	5.61	0.0001
WG g/fish	324.00^c^	383.50^b^	440.50^a^	3.17	0.0001
SGR,%/day	0.54^c^	0.60^b^	0.67^a^	0.002	0.0001
FCR	1.94^a^	1.87^b^	1.76^c^	0.008	0.0001
Survival rate, %					
Survival rate, %	83.34^b^	96.67^a^	100.00^a^	2.72	0.0443

Means followed by different letters in the same row are significantly different (*P ≤* 0.05)

*NWE* non-water exchange system: fish were reared under unchanged water

*CW* Clear water system: water exchange rate of 50% of tank volume weekly

*BFT* Biofloc technology system: addition of molasses daily for tank and without water exchange

*IBW* Initial body weight, *FBW* Final body weight, *WG* Weight gain

### Blood biochemical parameters

Cortisol, glucose, cholesterol, alanine aminotransferase (ALT), aspartate aminotransferase (AST), creatinine, and urea of males and females are showed in Table 4. BFT and CW systems are significantly (*P <* 0.05) affected the levels of cortisol, glucose, creatinine, and urea of males and females compared to NWE system. Lower values of cortisol, glucose, creatinine, and urea of both males and females were recorded in BFT and CW systems compared to NWE system. Also BFT and CW recorded higher values of cholesterol, ALT, and AST of males and females compared to NWE system.

**Table 4:** Blood parameters of African catfish (*Clarias gariepinus*) male brood-stock and female under different rearing conditions

Parameters	Rearing systems			± SEM	*P*-value
	NWE	CW	BFT		
Male					
Cortisol, mg/dl	60.50^a^	47.50^b^	43.00^b^	1.56	0.0191
Glucose, mg/dl	165.50^a^	154.50^ab^	147.00^b^	1.99	0.0833
Cholesterol, mg/dl	233.00^c^	282.00^b^	369.50^a^	6.40	0.0062
AST,U/L	197.00^b^	258.50^a^	290.50^a^	7.48	0.0071
ALT,U/L	112.50^c^	127.00^b^	138.50^a^	1.70	0.0104
Creatinine, mg/dl	0.70^a^	0.57^ab^	0.51^b^	0.03	0.0936
Urea, mg/dl	10.92^a^	8.26^b^	7.30^b^	0.33	0.0372
Female					
Cortisol, mg/dl	54.50^a^	41.50^b^	39.50^b^	1.56	0.0384
Glucose, mg/dl	143.00^a^	136.00^b^	129.50^b^	1.99	0.0203
Cholesterol, mg/dl	248.00^c^	335.50^b^	432.00^a^	6.40	0.0011
AST,U/L	168.00^c^	198.00^b^	222.50^a^	7.48	0.0124
ALT,U/L	81.50^b^	88.50^b^	108.00^a^	1.70	0.0103
Creatinine, mg/dl	0.65	0.50	0.46	0.03	0.1382
Urea, mg/dl	9.04^a^	7.04^b^	6.04^b^	0.33	0.0264

Means followed by different letters in the same row are significantly different (*P <* 0.05)

*NWE* Non-water exchange system: fish were reared under unchanged water

*CW* Clear water system: water exchange rate of 50% of tank volume weekly

*BFT* Biofloc technology system: addition of molasses daily for tank and without water exchange

### Sexual hormones

Testosterone, estrogen and progesterone of catfish brood-stock are presented in Table 5. BFT system is significantly (*P <* 0.05) improved the levels of testosterone and progesterone. The highest levels of testosterone and progesterone were recorded in BFT system compared to CW and NWEsystems. The highest level of estrogen was recorded in NWE system.

**Table 5:** Hormones Parameters of African catfish (*Clarias gariepinus*) brood-stock under different rearing conditions

Parameters	Rearing systems			± SEM	*P*-value
	NWE	CW	BFT		
Testosterone, ng/ml (male)	3.24^c^	3.89^b^	4.75^a^	0.12	0.0084
Estrogen, ng/ml (female)	4,093.50^a^	3,671.00^b^	3,481.00^b^	87.17	0.0342
Progesterone, ng/ml (female)	1.48^c^	2.73^b^	3.52^a^	0.13	0.0043

Means followed by different letters in the same row are significantly different (*P <* 0.05)

*NWE* Non-water exchange system: fish were reared under unchanged water

*CW* Clear water system: water exchange rate of 50% of tank volume weekly

*BFT* Biofloc technology system: addition of molasses daily for tank andwithout water exchange

### Reproduction parameters of catfish female brood-stock

Absolute fecundity (AF), relative fecundity (RF), gonado somatic index (GSI), and egg diameters of catfish female brood-stock are exhibited in Table 6. Significant differences (*P <* 0.05) were observed in catfish female brood-stock among different treatments. The highest AF, RF, GSI and egg diameter of catfish female brood-stock were recorded in BFT system compared to CW and NWE systems.

**Table 6:** Reproductive parameters of African catfish (*Clarias gariepinus*) under different rearing conditions

Parameters	Rearing systems			± SEM	*P*-value
	NWE	CW	BFT		
female weight, g/fish	680.00^c^	743.50^b^	799.00^a^	5.61	0.0001
Absolute fecundity eggs/female	71,685.00^c^	94,232.50^b^	118,154.00^a^	707.85	0.0343
Relative fecundity eggs/g female	101.72^c^	122. 90^b^	148.21^a^	1.09	0.0001
Gonadosomatic index, %	6.34^b^	9.83^a^	11.84^a^	0.53	0.0001
Egg diameter, mm	0.85^b^	0.96^ab^	1.02^a^	0.03	0.0125

Means followed by different letters in the same row are significantly different (*P <* 0.05)

*NWE* Non-water exchange system: fish were reared under unchanged water

*CW* Clear water system: water exchange rate of 50% of tank volume weekly

*BFT* Biofloc technology system: addition of molasses daily for tank andwithout water exchange

### Larval production and growth parameters

Number of larvae/female and number of larvae/g female, larvae weight, larvae length and larvae survival (after 20 days growth) of African catfish female brood-stock in different treatments are showed in Table 7. Significant differences (*p ≤* 0.05) were found in different larvae parameters among different treatments. The highest number of larvae/female, number of larvae/g female, larvae weight, larvae length and larvae survival of African catfish female brood-stock were recorded in BFT system compared to CW and NWE systems.

**Table 7:** Larval production and growth parametersof African catfish (*Clarias gariepinus*) under different rearing conditions

Parameters	Rearing systems			± SEM	*P*-value
	NWE	CW	BFT		
No. of larvae/female	50,910.00^c^	60,072.50^b^	66,965.00^a^	774.84	0.0025
No. of larvae/g female	72.25^c^	78.35^b^	83.99^a^	0.86	0.0062
Larvae weight, mg/larvae^*^	34.05^b^	50.25^a^	57.05^a^	3.25	0.0334
Larvae length, mm/larvae^*^	16.50^b^	18.50^ab^	20.50^a^	0.50	0.0251
Larvae survival, %^*^	29.25^b^	39.75^a^	46.00^a^	2.09	0.0001

Means followed by different letters in the same row are significantly different (*P <* 0.05)

*NWE* Non-water exchange system: fish were reared under unchanged water

*CW* Clear water system: water exchange rate of 50% of tank volume weekly

*BFT* Biofloc technology system: addition of molasses daily for tank andwithout water exchange

^*^Weight, length and survival rate of larvae after 20 days

## Discussion

In general, good water quality reflects good health and reproduction of fish species. Biofloc system maintains water quality parameters to confirm environmental stability when brood-stock reared in the system [90, 115]. Heterotrophic bacteria and other probiotic in biofloc system positively affect water quality by controlling propagation pathogenic and stimulating the colonization of valuable bacteria, which can afford various health-promoting effects in brood-stock [41, 60, 66, 71, 89, 107]. The water quality analysis in the present study indicated that all parameters using biofloc technology system remained within concentrations suitable for *Clarias gariepinus* culture (Boyed, [17]). Also it exhibited lower values of ammonia and nitrite in BFT system compared to other systems (CW and NWE), this explain the positive effect of biofloc system for reducing the harmful nitrogen [12, 28]. In line with our finding biofloc treatment maintained good water quality for striped catfish (*Pangasianodon hypophthalmus*) by reduced total ammonia and nitrite [35]. In addition, Popoola et al. [94] indicated that the decrease of ammonium and nitrite levels in the biofloc system likely contributes to improved growth performance in *C. gariepinus*. They also added that the possible assistances of biofloc technology are multifaceted and involve as well in keeping good water quality, that minimizes stress, as well as enhance biosecurity and possibly reducing disease occurrence. Additional contributing factor may be the utilization of bioactive compounds in the biofloc, which enhances the nutritional status and immunity of the aquatic animals versus diseases [33, 114]. There is a positive relation between growth performance and water quality. In this context Dauda et al., [22] suggested that the better growth may also be credited to the combined effects of improved water quality and increased densities of bacteria and zooplankton. Diatin et al. [27] demonstrated that catfish farming using biofloc technology could be a viable alternative for the future development of fish farming due to its efficient use of water. It can conserve water up to 14 times more than traditional water exchange systems, which require a 50–75% daily water exchange [22].

Biofloc is a good media for the growth of different types of zooplankton resulting the abundant of nutrients in biofloc system [105]. Protozoa, rotifer, copepods and oligochaete are the most zooplankton groups were presented in the biofloc system [14, 40]. The present study found different zooplankton groups including protozoa, rotifer, copepods in different treatments, while the highest count of zooplankton were found in BFT system compared to CW and NWE systems. Our results are in agreement with Said and Taha [99] who indicated their finding of higher zooplankton count in all biofloc treatments (different densities) of Nile tilapia *Oreocromis niloticus* when compared with the clear system. The abundance of zooplankton might be attributed to the presence of biofloc which established a proper environment for zooplankton growth. Similar findings were reported by Avnimelech [11], Emerenciano et al. [39] and Gao et al. [48]. Popoola et al. [94] confirmed that the production and accumulation of biofloc can serve as an essential food source for zooplankton and consequently enhances the growth of *C. gariepinus*. The presence of several live feeds such as rotifer, protozoa, and copepod can boost the growth performance of *Clarias gariepinus*. Similarly, Loureiro et al. [74] credited the good performance achievement of *Litopenaeus vannamei* in biofloc systems to the availability of diverse live feeds. Hosain et al. [63] demonstrated that the richness of zooplankton provided some additional nutrition to *M. rosenbergii* which decreased the feed conversion ratio and enhanced economic outcomes.

Our study demonstrated that biofloc technology enhanced the growth of African catfish (*C. gariepinus*). The results observations revealed an increase in the weight of catfish cultured using the BFT system compared to CW and NWE systems. Former studies have demonstrated that fish reared using biofloc technology exhibit superior growth and survival rates compared to those reared without biofloc, specifically in African catfish, *Clarias gariepinus* [25, 35]. The enhanced growth observed in the current study using biofloc treatment can be attributed to several factors inherent to the biofloc system. One major factor is the richness of active heterotrophic bacteria, which can integrate waste nitrogen from uneaten feed and fish feces, generating new cellular protein with an optimal amino acid profile for fish consumption [12, 20, 113]. Moreover, Popoola et al. [94] explained that this ongoing nutrient recycling provides the fish with a steady supply of high-quality protein, thereby enhancing their growth. Studies have shown that carbohydrate addition can lead to the production and accumulation of biofloc [11], which serves as an essential food source for zooplankton and consequently enhances the growth of *C. gariepinus* [94]. Additionally, Gallardo-Colli et al. [47] found that the abundance of microorganisms in biofloc systems was higher. Also, the results of Alinsangao et al. [4] indicated that African catfish in biofloc-based tanks were less dependent on supplemental feeds and likely utilized alternative food sources, leading to significantly higher growth performance. In line with our finding survival rate of striped catfish significantly enhanced when reared in biofloc system, while the enhancement may be due to the positive effect of immune stimulant compounds and antioxidant in biofloc system [13, 112].

Blood characteristics can serve as indicators to assess physiological responses in fish [57]. The decrease of cortisol, glucose, creatinine, and urea values in our study can be attributed to the less stressful conditions of the CW and BFT systems. While ALT and AST levels were high in biofloc system in the present study. In contrast with our results Adineh et al. [3] found lower values of ALT and AST in carp fish reared under biofloc system, indicating lesser stress condition. The higher levels of ALT and AST in the present study in biofloc treatment may be due to the higher protein intake from diet (30% cp) beside the microbial protein from biofloc. In this context Melo et al. [81] found higher ALT and AST in *Rhamdia quelen* when fed diet high in protein where the fish use of excess dietary amino acids for growth, as well as substrate for gluconeogenesis, particularly for ALT activities. Moreover, Meton et al. [82] found higher levels of ALT an AST in *Sparus aurata* when fed diet contain higher protein/carbohydrate ratio, these may be occurred in biofloc system as a result to carbon addition in the system and presence of microbial protein as additional protein source in biofloc [12]. The changes of hormone levels of cortisol, blood glucose can be indicators for the stress responses in animals [58]. Moreover, cholesterol level significantly increased with BFT treatments. The impact of stressors on fish has been allied to a decline in body lipid content [106]. Its levels in blood serum have been associated to stress controlling [76, 93]. This may explain that the fish exposed to less stressful environment like the biofloc condition keep higher level of blood lipids as cholesterol when compared to the CW and NWE systems. Said and Taha [99] observed the same results in Nile tilapia which showed higher blood cholesterol levels in biofloc treatment. It was concluded that Biofloc technology can maintain water quality in conditions that meet the requirements of *C. gariepinus* [58], which creates healthier environment for cultured fish.

Higher levels of steroid hormones like testosterone, estrogens, and progesterone are indicators to gonadal development and maturation of ovaries and testicles in fish [80, 91]. The present study exhibited higher levels of testosterone and progesterone in brood stock reared in biofloc system. In line with our finding red tilapia brood-stock reared in water salinity 18 ppt under biofloc condition has higher levels of testosterone and progesterone [100]. Moreover, Manzoor et al. [78] proved the positive effects of bioflocs on gonadal development in common carp, which was linked to higher blood cholesterol levels. Cholesterol is recognized as an originator for steroid hormones, including testosterone, estrogens, and progesterone [75]. An elevation in blood cholesterol levels indicates improved sexual maturation in brooders, as observed in Nile tilapia [32]. The observed elevation of testosterone and progesterone along with the decrease of estrogen in biofloc treatments in our study can be attributed to complicated interactions involving stress resistance, nutritional intake, immune response, reproductive performance and environmental circumstances related to biofloc systems. The reduction of estrogen in our study may be coupled with the onset of oocyte maturation/ovulation where, estrogen level, is low in the postvitellogenic ovary, in several species including the catfish (Joy et al. [64]). Further research would be required to identify the exact mechanisms involved.

Food availability is an important factor for enhancing the gonadal maturation and spawning of different fish species [42]. In this regard, biofloc is a possible readily available food source at all times in fish ponds, where it contains variety of nutrients representing in microbial protein and different types of microorganisms (zoo and phytoplankton, heterotrophic/chemoautotrophic bacteria, filamentous cyanobacteria, dinoflagellates, and nematodes) [40, 66]. The last components assumed amino acids, lipid and fatty acids which are important for fish reproduction [42, 46, 73]. Recent several studies have explored the impact of biofloc system on the reproductive performance and physiology of numerous cultured aquatic species [6, 32, 35, 37, 65]. The present study found that higher fecundity (absolute fecundity and relative fecundity), gonado somatic index and egg diameter in BFT systems compared to other systems (CW and NWE). In consistent with the present study Sallam et al. [100] found higher values of egg number per gram, ovary weight, absolute fecundity and relative fecundity in red tilapia brood-stock reared in water salinity 18 ppt under biofloc condition compared to clear condition. Furthermore, Ekasari et al. [35] stated that the absolute fecundity on day-122 of biofloc broods-tocks was 26% higher than that of the clear system brood-stock in their experiment (*P <* 0.05). This might be explained by the good quality of biofloc which noticeably nutritious as a food source for the fish. Ekasari et al. [32] assessed the reproductive performance of Nile tilapia brooders in a BFT system compared to clear system. Their results indicated that more fry were significantly produced by broodstock reared in the biofloc system, which was linked to elevated blood glucose and total cholesterol amounts. These outcomes suggest that biofloc system can enhance the reproductive performance of brooders by boosting the availability of energy and essential nutrients for instance, cholesterol as the primary creator of steroid hormones, potentially resulting in higher harvests and better quality of offspring [68]. Manzoor et al. [78] proved the positive effects of bioflocs on gonadal development in common carp. Females in the BFT treatment displayed advanced ovary maturity phases, the highest absolute fecundity, as well as GSI (24.47% vs. 17.97%). Cardona et al. [19] explained that the improved reproductive performance of *Litopenaeus stylirostris* in biofloc systems might be attributed to the biofloc particles providing vital nutrients for reproduction, such as antioxidants (glutathione), as well as essential lipids. Biofloc contains 12 to 50% protein, 0.5 to 41% lipids, 14 to 59% carbohydrates, and 3 to 61% ash in a dry weight basis [14, 36]. Also biofloc contain fatty acids like, linoleic acid (LA, 3.58 mg/g dry weight), α-linolenic acid (ALA, 0.46), arachidonic acid (ARA, 0.40), eicosapentaenoic acid (EPA, 1.67), docosahexaenoic acid (DHA, 0.29 mg/g dry weight) [35]. The last nutrients in biofloc may be contributed in the enhancement of reproduction performance of different fish species reared in biofloc system. In this sense, fatty acids positively affected the fecundity of different fish species especially n-3 HUFA and n-6 PUFA by improving the spawning frequency and fry production [15, 111]. Several studies found higher PUFA in biofloc content especially EPA and DHA [19, 35], (Ekasari et al., [31]), while EPA and DHA which are required to support gonadal maturation and high egg quantity and quality (Dabrowski and iereszko [23]). Moreover, EPA and DHA play vital role in improving reproduction performance in fish such as egg and larvae development, fecundity and brood-stock healthy in general [70].

Quality of brood-stock reflects the quality of their larvae survival where there is a relation between them [19, 52, 118]. Biofloc system achieved the last purpose where the quality of catfish larvae and survival rate were improved through the elevation of embryonic development rate [35]. The present study exhibited higher larvae number, weight, length and survival in BFT systems compared to other systems (CW and NWE). In harmony with our results Ekasari et al. [35] exhibited higher survival rate of catfish larvae from brood-stock reared in biofloc system. As well as, Ekasari et al. [34] found higher survival rate of Nile tilapia larvae reared in biofloc system compared to clear system. The improvement of larvae quality may be due to the positive effect of biofloc through the microorganisms which act as immune stimulator and protector from stress condition [68, 98]. Also, the strength of larvae coming from biofloc brood-stocks were approved by their better survival rates. It can be proposed that the good impact of bioflocs on larval strength observed in our study is due to an increase in maternally resulting immune defense which might be transferred to the larvae and caused an enhancement of larval tolerance to environmental pressure. As per Heinecke et al. [61] and Ghaedi et al. [49], the immune system of recently hatched fish larvae in their early stage of life is not completely established, making them heavily dependent on maternally transferred immunity. Ekasari et al. [35] indicated that the stimulated immunity in biofloc system, which resulted through experience a microbe-rich environment or in situ consumption of bioflocs, may enhance the immunity of brood-stock reared in biofloc systems and the boosted immunity could then be transferred maternally, leading to improved immunity of the larvae. As author knowledge there are a limitation in histological or immunological assessments of the organs of brood-stock fish under biofloc system, so future studies should be determining the histological or immunological assessments of brood–stock of different fish species under biofloc system.

## Conclusion

In conclusion, it was confirmed in our study that rearing African catfish brood-stock in a biofloc system, with its efficient use of water resulted in superior growth performance and better reproductive traits comparing to that of the CW and NWE systems. These findings support the potential of BFT as a viable strategy to enhance catfish brood-stock productivity. Future studies are recommended to address this important point in the future perspectives for enhancing the reproduction performance of marine fish and also improving the survival rate of the larvae of marine fish through BFT system.

## Data Availability

Data will be made available on request.
